# The Effect of Enzymolysis on Performance of Soy Protein-Based Adhesive

**DOI:** 10.3390/molecules23112752

**Published:** 2018-10-24

**Authors:** Yantao Xu, Yecheng Xu, Yufei Han, Mingsong Chen, Wei Zhang, Qiang Gao, Jianzhang Li

**Affiliations:** 1 Key Laboratory of Wood Material Science and Utilization, Beijing Forestry University, Beijing 100083, China; xuyantao@bjfu.edu.cn (Y.X.); xuyecheng@bjfu.edu.cn (Y.X.); hanyufei96@163.com (Y.H.); chen_bjfu@163.com (M.C.); zhangwei@bjfu.edu.cn (W.Z.); 2 Beijing Key Laboratory of Wood Science and Engineering, Ministry of Education, College of Materials Science and Technology, Beijing Forestry University, Beijing 100083, China

**Keywords:** soy protein isolate, bromelain, triglycidylamine, viscosity, water resistance, adhesive

## Abstract

In this study, bromelain was used to break soy protein molecules into polypeptide chains, and triglycidylamine (TGA) was added to develop a bio-adhesive. The viscosity, residual rate, functional groups, thermal behavior, and fracture surface of different adhesives were measured. A three-ply plywood was fabricated and evaluated. The results showed that using 0.1 wt% bromelain improved the soy protein isolate (SPI) content of the adhesive from 12 wt% to 18 wt%, with viscosity remaining constant, but reduced the residual rate by 9.6% and the wet shear strength of the resultant plywood by 69.8%. After the addition of 9 wt% TGA, the residual rate of the SPI/bromelain/TGA adhesive improved by 13.7%, and the wet shear strength of the resultant plywood increased by 681.3% relative to that of the SPI/bromelain adhesive. The wet shear strength was 30.2% higher than that of the SPI/TGA adhesive, which was attributed to the breakage of protein molecules into polypeptide chains. This occurrence led to (1) the formation of more interlocks with the wood surface during the curing process of the adhesive and (2) the exposure and reaction of more hydrophilic groups with TGA to produce a denser cross-linked network in the adhesive. This denser network exhibited enhanced thermal stability and created a ductile fracture surface after the enzymatic hydrolysis process.

## 1. Introduction

Biomass adhesives, such as tannin, lignin, carbohydrate, unsaturated oil, and protein-based adhesives, have been widely studied as alternatives to formaldehyde-based adhesives to eliminate formaldehyde hazard in wood panels [1]. Among these biomass adhesives, the soy protein adhesive is a rich, formaldehyde-free, low-cost raw material and exhibits considerable potential for development [2]. However, poor water resistance limits the application of soy protein adhesives [3]. Most studies have focused on using chemical modification to improve the performance of soy protein-based adhesives [4], such as denaturing agent modification [5], graft modification [6], biomimetic modification [7], latex modification [8], and synthetic resin modification [9]. Polyacrylamide and epoxide have been proven to be effective as cross-linkers for soy protein-based adhesives, with the resultant plywood meeting the requirements for interior plywood [10,11]. However, these modified adhesives have a low solid content and high viscosity, resulting in a panel that is difficult to apply and has poor production stability.

Wood is a porous material. The bond strength of a wood panel mainly comes from mechanical interlocking after the curing process of the adhesive [12]. The high molecule weight of the soy protein-based adhesive impedes its penetration into the wood, thus barely forming an interlock, resulting in a low bond strength and wood failure. In addition, soy protein is the aggregation of high-molecular-weight polypeptide chains with the complex quaternary structure, which implies numerous active groups in the interior of the protein, resulting in a low reactivity of protein. From another perspective, a high molecular weight of protein leads to a high viscosity and low solid content of the resultant adhesive, which means a lot water is introduced into the wood panel during the fabrication process, leading to poor production stability of the resultant panel.

In recent years, enzyme technology has gradually developed. Driving this new thrust are three major new goals, that is, maximizing the exploitation of renewable resources as sources of raw materials for the production of multifunctional polymers, development of an environmentally friendly process, and development of biodegradable products [13]. Enzyme technology has been widely used. For example, the use of enzymes for the selective hydrolysis/treatment of polymers and materials [14], the mild surface functionalization of polymers such as polyethylene terephthalate (PET) and polylactic acid (PLA), and the subsequent coupling of molecules and grafting of molecules on wood after enzymatic pre-treatment has achieved certain results [15,16]. The enzyme technology is becoming increasinly more mature. Enzyme modified soy protein is also a feasible method.

In the current study, a protein endonuclease–bromelain was used to break down protein molecules into polypeptide chains to reduce the viscosity and improve the solid content of the soy protein isolate (SPI) adhesive. The active groups on soy protein molecule chains were also exposed during this process. These polypeptide chains then reacted with a laboratory-made cross-linker triglycidylamine (TGA) to develop a soy protein-based adhesive. The effects of the low molecular weight of protein on the performance of the resultant adhesive, including the viscosity, residual rate and the functional groups, thermostability, and fracture surface, were characterized. Three-ply plywood samples were fabricated using the resultant adhesives, and their wet shear strengths were evaluated.

## 2. Materials and Methods 

### 2.1. Materials

**Figure 1:**
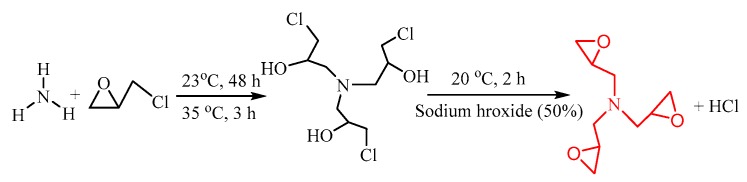
The synthesis procedure of cross-linker triglycidylamine (TGA).

SPI with 95% protein content was obtained from Yuwang Ecological Food Industry Co, Ltd. (Jinan, China) [17]. Poplar veneer (200 × 200 × 1.5 mm, 8% moisture content) from Hebei Province of China was provided. Bromelain (BR, 300 u/mg, CAS # 37189-34-7) was purchased from Shanghai Yuanye Group (Shanghai, China). Triethylamine, a laboratory-made epoxy cross-linker, was also used. The reaction pathway of TGA is systhsized following our previous research [18] and illustrated in Figure 1. Epichlorohydrin and aqueous ammonia with a mole ratio of 5:1 was placed into a three-necked flask equipped with a condenser and a stirrer. The mixture was stirred continuously at a rate of 800 rpm. Ammonium triflate was used to catalyze the reaction at 23 °C for 48 h, and then at 35 °C for 3 h. The residual epichlorohydrin and ammonium hydroxide were removed by a vacuum distillation, and the result was a colorless syrup consisting mostly of tris(3-chloro-2-hydroxypropyl) amine. An excess of sodium hydroxide solution (50%) was added for the epoxy-ring closure reaction at 20 °C for 2 h. Because the reaction was highly exothermal, an external ice-water cooling circulator was required to hold the temperature. The precipitate of sodium chloride was filtered off, and the residue was vacuum distilled to obtain pure viscous TGA.

### 2.2. Preparation of Soy Protein Adhesive

Protein and water were mixed to develop an SPI adhesive (Table 1). Bromelain was added to the SPI adhesive and then stirred in a water bath at 50 °C for 20 min, allowing full digestion. The mixture was then placed in a water bath at 90 °C and then stirred for 10 min to deactivate bromelain. TGA was ultimately added into the mixture to develop the final adhesives.

### 2.3. Preparation and Evaluation of Plywood

Three layers of poplar plywood were prepared in this study. The adhesive was evenly coated on both sides of the core veneer with glue spreading of 200 g/m^2^. The coated plywood was placed between two uncoated veneers, perpendicular to the grain of the adjacent veneer. The laminated plywood was hot-pressed at 120 °C and 1.0 MPa for 6 min, and two sheets of plywood were produced using the same adhesive formulation. The shear strength of the plywood was determined in accordance with the Chinese National Standard GB/T 17657 (2013) [19]. The prepared plywood was allowed to remain at room temperature for at least 24 h. Twelve specimens measuring 100 mm × 25 mm (glue area, 25 mm × 25 mm) were uniformly cut from the center and the edges of the two sheets of plywood.

### 2.4. Viscosity

The viscosity of the soybean adhesives was measured using a Brook field DV-II viscometer, employing the rotor with a spinning rate of 100 rpm. An average of three replicate measurements was reported as the viscosity of each sample.

### 2.5. Residual Rate Test

The adhesive sample was placed in an oven at 120 ± 2 °C until a constant weight was obtained and then ground into 100 mesh powder (0.15 mm) using a ceramic mortar. To determine mass loss, the cured adhesive was wrapped with a qualitative filter paper and then placed in a glass with distilled water [20]. After blistering for 6 h in an oven at 60 ± 2 °C, the sample was dried (120 ± 2 °C, 3 h) and weighed. The mass loss was determined by calculating the difference in weight before and after hydrolysis.

### 2.6. Wet Shear Strength Measurement 

In accordance with the Chinese National Standard (GB/T 17657-2013), the wet shear strength of the second-grade plywood (interior use plywood) was determined. Twelve plywood specimens (25 mm × 100 mm) were cut from two pieces of plywood, immersed in water at 63 °C for 3 h, dried at room temperature for 10 min, and subjected to tensile testing. Wet shear strength was calculated using Equation (1). The standard deviation of the data was calculated.

(1) $$\mathrm{Shear}\;\mathrm{strength}\;=\;\frac{\mathrm{Force}\;\left(N\right)}{\mathrm{Gluing}\;\mathrm{area}\;\left(m^{2}\right)}\;$$

### 2.7. Fourier Transform Infrared (FTIR) Spectroscopy 

The different adhesive samples prepared were cured in an oven at 120 ± 2 °C until a constant weight was obtained. Then, we ground the adhesive into a powder. FTIR spectra of the different cured adhesives were recorded using a Nicolet 7600 spectrometer (Nicolet Instrument Corporation, Madison, WI, USA) from 500 to 4000 cm^−1^ with a 4 cm^−1^ resolution using 32 scans.

### 2.8. Thermogravimetric (TG)

The different adhesives were cured in an oven at 120 ± 2 °C until a constant weight was obtained, and we then ground the adhesive into a powder. The thermal stabilities of the cured adhesive samples were tested using a TGA instrument (TA Q50, Waters Company, Milford, MA, USA). Approximately 5 mg powdered samples were weighed in a platinum cup and scanned from 30 to 600 °C at a heating rate of 10 °C min^−1^ in a nitrogen environment while recording the weight change. 

### 2.9. Scanning Electron Microscopy (SEM) 

The fracture surface micrographs of cured adhesives were measured using a JSM-6500F field emission scanning electron microscope (FE-SEM) (JEOL USA Inc., Peabody, MA, USA). Prior to testing, the fracture surface was placed on an aluminum stub and a 10 nm gold film was coated on using an ion sputter (HITACHI MCIOOO, Tokyo, Japan).

## 3. Results and Discussion

### 3.1. Viscosity

Extremely high viscosity rendered the coating process difficult and ineffective; meanwhile, extremely low viscosity led to the over penetration of adhesive into the wood surface in the manufacture of plywood [21]. A wood adhesive requires a suitable viscosity to ensure strong contact with wood. In addition, it should exhibit adequate penetration and mechanical interlocking with the substrates [22]. 

Table 2 shows that the native SPI adhesive (adhesive 0) contains 12 wt% of SPI; the viscosity is 61,000 cP, which presents no flowability in the adhesive. As a protein endonuclease, bromelain broke soy protein molecules into polypeptide chains, reducing the molecular weight of the soy protein and exhibiting low viscosity. When the SPI content was increased to 18 wt%, the development of a uniform pure soy protein adhesive was impeded. However, when using bromelain in the adhesive formulation, the viscosity of adhesive (1) reached 62,880 cP, which was similar to that that of adhesive (0). This similarity indicated that enzymatic hydrolysis effectively increased the solid content of the soy protein adhesive. Further addition of TGA into the adhesive treated with enzymatic hydrolysis led to a gradual decrease in viscosity from 62,880 cP to 285 cP. With the addition of 3 wt% TGA into the adhesive formulation, the viscosity of adhesive (2) decreased by 48.64% relative to that of adhesive (1). Further addition of TGA to 12 wt% caused a reduction in the viscosity of adhesive (5) by 99% to 285 cP. This decrease was attributed to the low molecular weight of TGA, which reduced the friction in the decomposed soy protein macromolecules, consequently decreasing the viscosity of the adhesive. However, the viscosity of adhesive (5) was too low, such that the adhesive could easily over penetrate the wood surface during gluing, preventing the formation of an adhesive layer. As a control, adhesive (6) contained 18 wt% SPI and 9 wt% TGA, and its viscosity was 2200 cP, which was 211% higher than that of adhesive (4). This result also indicated that enzymatic hydrolysis effectively decreased the viscosity of the adhesive. 

### 3.2. Residual Rate Test 

When the flour of the cured adhesive was immersed in water, the amount of insoluble mass determined the cross-link density and water resistance of the adhesive [23]. The bond strength of the native SPI adhesive was mainly based on the hydrogen bond between active groups. Owing to the hydrophilicity of most active groups, SPI adhesive (0) exhibited poor water resistance. 

**Figure 2:**
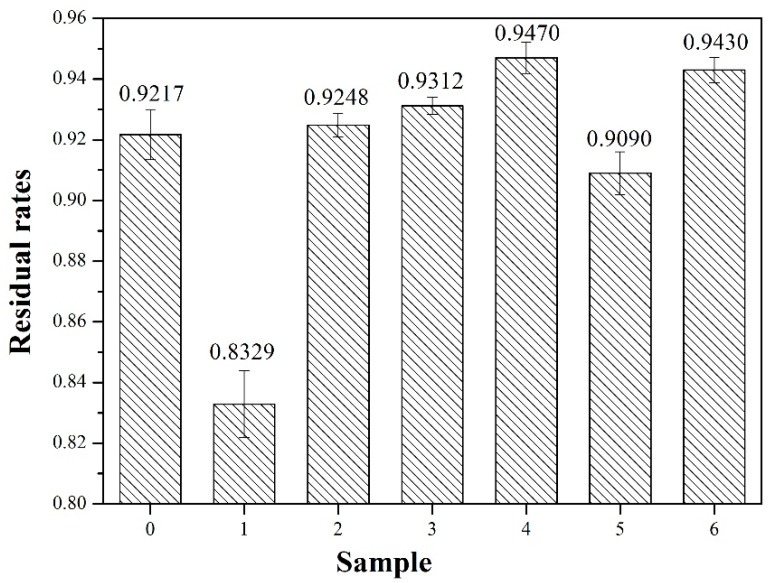
Residual rates of different adhesive samples: 0 (12 wt% soy protein isolate (SPI)), 1 (18 wt% SPI/bromelain), 2 (18 wt% SPI/bromelain/3 wt% TGA), 3 (18 wt% SPI/bromelain/6 wt% TGA), 4 (18 wt% SPI/bromelain/9 wt% TGA), 5 (18 wt% SPI/bromelain/12 wt% TGA), and 6 (18 wt% SPI/9 wt% TGA).

Figure 2 showed that the residual rate of SPI adhesive (0) was 92.17%. After enzymatic hydrolysis, the residual rate of adhesive (1) decreases to 83.29%, indicating a reduction in the water resistance of the adhesive. This effect was attributed to the following reasons: First, bromelain reduced the molecular weight of soy protein by breaking down protein into molecular chains. This process produced small soy protein molecules, which were easier to dissolve in water. Second, enzymatic hydrolysis exposed the hydrophilic groups of the protein, such as –NH_2_, –COOH, which further reduced the water resistance of the SPI adhesives. When 3 wt% TGA was added to the adhesive formulation, the residue rate of the adhesive increased by 4.75% relative to that of adhesive (1). With a further increase in TGA addition to 9 wt%, the residual rate increased by 13.70% to the maximum value, which was higher than that of adhesive (6). This effect was attributed to the cross-linking of the epoxy groups of TGA with the exposed active groups of soy protein chains, as well as the formation of a more compact cross-linked network structure, resulting in improved water resistance of the adhesive. With an increase in the number of epoxy groups, more cross-linking reactions led to the formation of a more compact cross-linked network structure. However, when TGA reached 12 wt%, the residue ratio decreased by 4% to 90.90%, indicating excessive TGA dosage. Soluble TGA was eluted from the filter paper, resulting in the reduction of the residue ratio. 

### 3.3. Wet Shear Strength Measurement 

**Figure 3:**
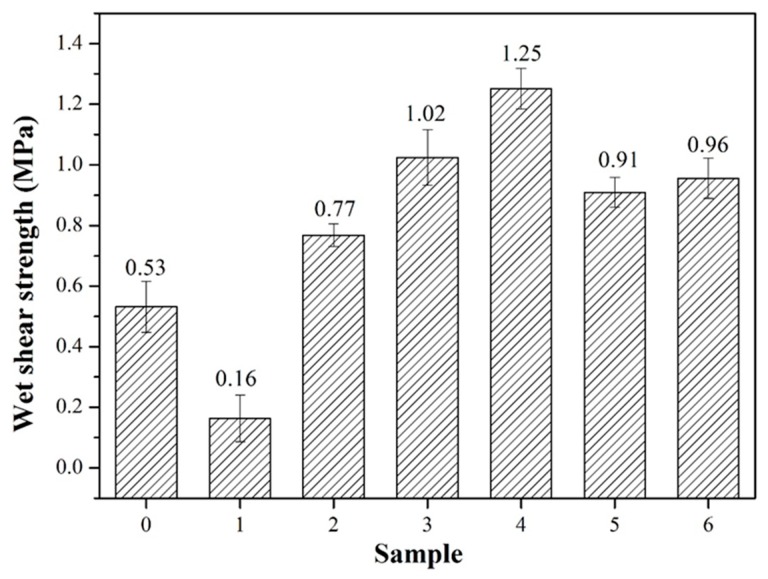
Wet shear strength of the different adhesive samples: 0 (12 wt% SPI), 1 (18 wt% SPI/bromelain), 2 (18 wt% SPI/bromelain/3 wt% TGA), 3 (18 wt% SPI/bromelain/6 wt% TGA), 4 (18 wt% SPI/bromelain/9 wt% TGA), 5 (18 wt% SPI/bromelain/12 wt% TGA), and 6 (18 wt% SPI/9 wt% TGA).

**Figure 4:**
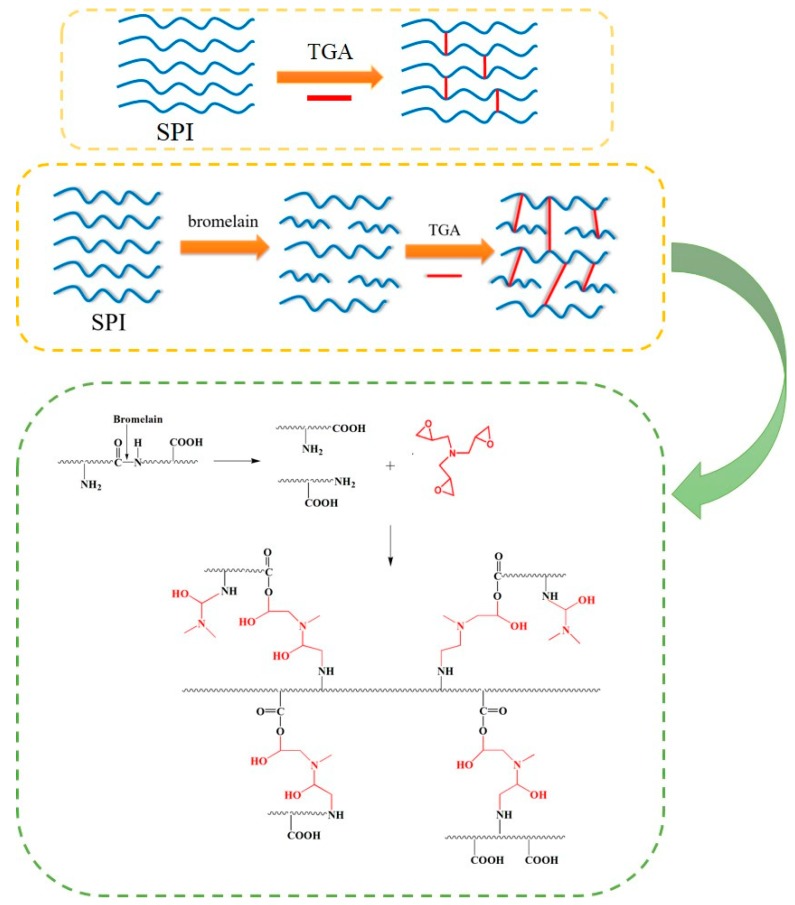
Cross-linking network of the soy protein adhesive.

Generally speaking, the wet shear strength of interior plywood required over 0.7 MPa according to the Chinese national standard. The wet shear strength of the plywood bonded by commercial adhesives are ranged from 0.7 to 1.2 MPa. The wet shear strength of the different adhesive samples is shown in Figure 3. The bond strength of the native SPI adhesive (adhesive 0) primarily resulted from the intermolecular hydrogen bond of soy protein, which was easily broken by moisture [24]. Thus, the wet shear strength of the plywood bonded with adhesive (0) was 0.53 MPa. After enzymatic hydrolysis, the wet shear strength of the plywood bonded with adhesive (1) decreased by 69.8% to 0.16 MPa. The 1, 2 level structures of protein are important for the bond strength formation of the protein adhesive. When the 1, 2 level structure was broken by bromelain, the bond strength was markedly reduced. When 3 wt% TGA was added to the adhesive formulation, the wet shear strength of the plywood bonded with adhesive (2) increased by 381% relative to that of adhesive (1). With a further increase in TGA addition to 9 wt%, the wet shear strength of the plywood reached 1.25 MPa, which increased by six times compared with that of adhesive (1). When TGA was added to the SPI-based adhesives, cross-linking occurred between the epoxy group and the reactive group (–NH_2_, –COOH), followed by the replacement of the weak hydrogen bond with a stable chemical bond. Simultaneously, a compact cross-linked network structure and a rigid curing system were formed, which improved the wet shear strength of the resultant plywood. As a control, the wet shear strength of the plywood bonded with adhesive (6) was 0.96 MPa, which was 30.2% lower than that of adhesive (4). This result could be attributed to the following reasons: First, the SPI molecule was degraded to polypeptide chains by bromelain, which exposed more active groups and produced more reactive sites to increase the reactivity of the adhesive, resulting in a denser cross-linked network structure formation and an increase in the water resistance of the adhesive. Second, with enzymatic hydrolysis, the viscosity of the adhesive was markedly reduced, and the permeability of the adhesive was improved. These changes led to enhanced mechanical interlocking with the wood formed, further improving the wet shear strength of the adhesive. Third, enzymatic hydrolysis also improved the solid content of the adhesive, which helped to improve the adhesive bond performance. A schematic of the adhesive reaction is presented in Figure 4. With the addition of 12 wt% TGA, the wet shear strength of the plywood was reduced to 0.91 MPa. This reduction was attributed to the considerably low viscosity of the adhesive, which led to the overpenetration of the wood surface and a reduction in the bond strength of the plywood.

### 3.4. Fourier Transform Infrared (FTIR) Spectroscopy Analysis

**Figure 5:**
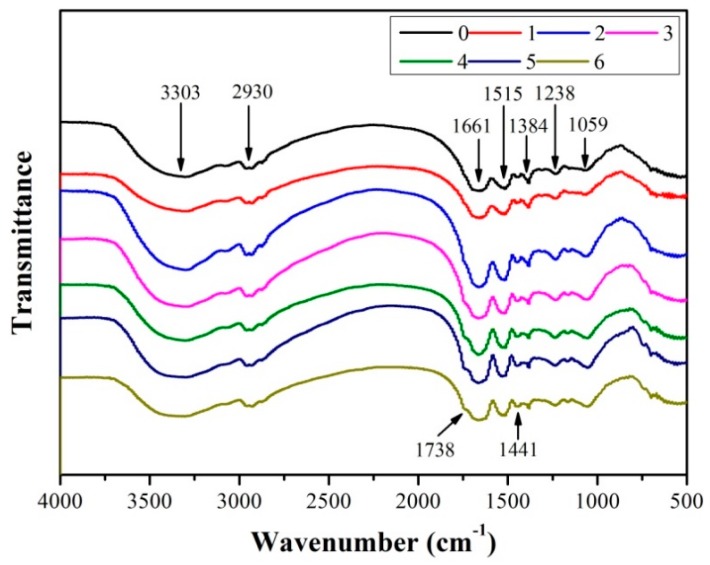
Fourier-transform infrared spectra of the different adhesive samples: 0 (12 wt% SPI), 1 (18 wt% SPI/bromelain), 2 (18 wt% SPI/bromelain/3 wt% TGA), 3 (18 wt% SPI/bromelain/6 wt% TGA), 4 (18 wt% SPI/bromelain/9 wt% TGA), 5 (18 wt% SPI/ bromelain/12 wt% TGA), and 6 (18 wt% SPI/9 wt% TGA).

Figure 5 presents the Fourier transform infrared spectra of the adhesives. The corresponding bending vibrations of free and bound N–H and O–H groups were approximately located at 3303 cm^−1^, which formed hydrogen bonds with the carbonyl group of the peptide linkage in soy protein [25]. The peak observed at about 2930 cm^−1^ was attributed to the symmetric and asymmetric stretching vibrations of the –CH_2_ group in the different adhesives [26]. In all adhesives, three characteristic bands of amides, namely, C=O stretching (amide I); N–H bending (amide II); N–H in-plane vibrations and the C–N stretching vibration (amide III), were observed at 1661, 1515, and 1238 cm^−1^, respectively [27]. The peaks at 1441 and 1384 cm^−1^ were the –CH_2_ deformation vibrations of the methyl group and the COO– stretching vibration, respectively [28]. 

In the mixed adhesives, the C–O bending absorption peak at 1059 cm^−1^ increased gradually with an increase in TGA addition. This result indicated that the TGA was well distributed in the adhesive system. No new peaks of the epoxy groups were found around 910 cm^−1^ after adding TGA into the adhesive formulation, indicating that the epoxy groups reacted with the active groups. With an increase in TGA addition, the peak of COO– at 1384 cm^−1^ gradually decreased, and a new peak of the carbonyl group gradually appeared at 1738 cm^−1^, which resulted from the esterification between the epoxy group and the carbonyl group of soy protein molecules. This finding was consistent with previous studies [29]. In addition, the soy protein adhesive contained numerous amino groups (–NH_2_) because the activation energy of the epoxy group with the amino group reaction was lower than that of the epoxy group with the carbonyl group reaction. TGA reacted faster with the amino groups in the soy protein molecules, indicating the occurrence of cross-linking. The peak of the C–O group at 1059 cm^−1^ in adhesive (4) was lower than that of adhesive (6), which might have resulted from the cross-linking of more TGA with reactive groups and the formation of a more compact cross-linked network structure. The cross-linking reaction between the TGA and the active groups led to the conversion of weak hydrogen bonds in the soy protein to rigid chemical bonds. This conversion reduced the number of hydrophilic groups and increased the cross-link density, thereby improving the water resistance of the soy protein adhesive.

### 3.5. Thermogravimetric (TG) Analysis 

**Figure 6:**
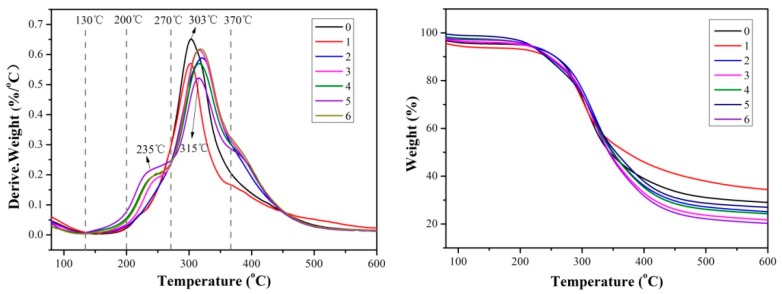
Thermogravimetric (TG, **left**) and derivative thermogravimetric (DTG, **right**) curves of the different adhesive samples: 0 (12 wt% SPI), 1 (18 wt% SPI/bromelain), 2 (18 wt% SPI/bromelain/3 wt% TGA), 3 (18 wt% SPI/bromelain/6 wt% TGA), 4 (18 wt% SPI/bromelain/9 wt% TGA), 5 (18 wt% SPI/bromelain/12 wt% TGA), and 6 (18 wt% SPI/9 wt% TGA).

Figure 6 shows the thermogravimetric and derivative thermal gravimetric curves of various adhesives. The thermal degradation of the adhesive can be divided into three stages. The slight weight loss before the temperature reached 130 °C was attributed to the evaporation of the residual moisture in the adhesive samples. The first stage was the post-reaction stage in the 130–200 °C temperature range. This was the result of the further curing reaction between SPI and the cross-linking agent, which produced vapor and gases, resulting in mass loss [30]. The second stage was the initial degradation stage in the 200–270 °C temperature range, which was attributed to the degradation of small molecules and the breakdown of some unstable chemical bonds. The third stage was the degradation phase of the framework structure in the 270–370 °C temperature range, which was caused by the degradation of the cross-linked network structure [31]. After the third degradation stage, further heating caused the breakdown of the C–C, C–N, and C–O linkages and the decomposition of soy protein backbone peptide bonds, which produced gases such as CO, CO_2_, NH_3_, and H_2_S [32,33].

Compared with the adhesives without TGA, the adhesives with TGA showed a new peak around 235 °C in the second stage. This new peak was improved with an increase in TGA addition, indicating that TGA reacted with active groups and that the structure of the adhesive changed. In the third stage, the peak of the adhesives with TGA shifted to a higher temperature, indicating an improvement in the thermal stability of the adhesives by forming a new structure. In the second and third stages, the degradation rate of adhesive (1) was lower than that of adhesive (0), indicating that the reduction in molecular weight by enzymatic hydrolysis improved the thermal stability of the adhesive. The peak degradation rate of adhesive (4) was lower than that of adhesive (6) in the third stage, also suggesting that enzymatic hydrolysis improved the thermal stability of the resultant adhesive. The cross-linking reaction between TGA and the enzymatic hydrolysis of soy protein molecules formed a more stable cross-linked network structure than that of the native soy protein adhesive.

### 3.6. Scanning Electron Microscopy (SEM) Analysis

**Figure 7:**
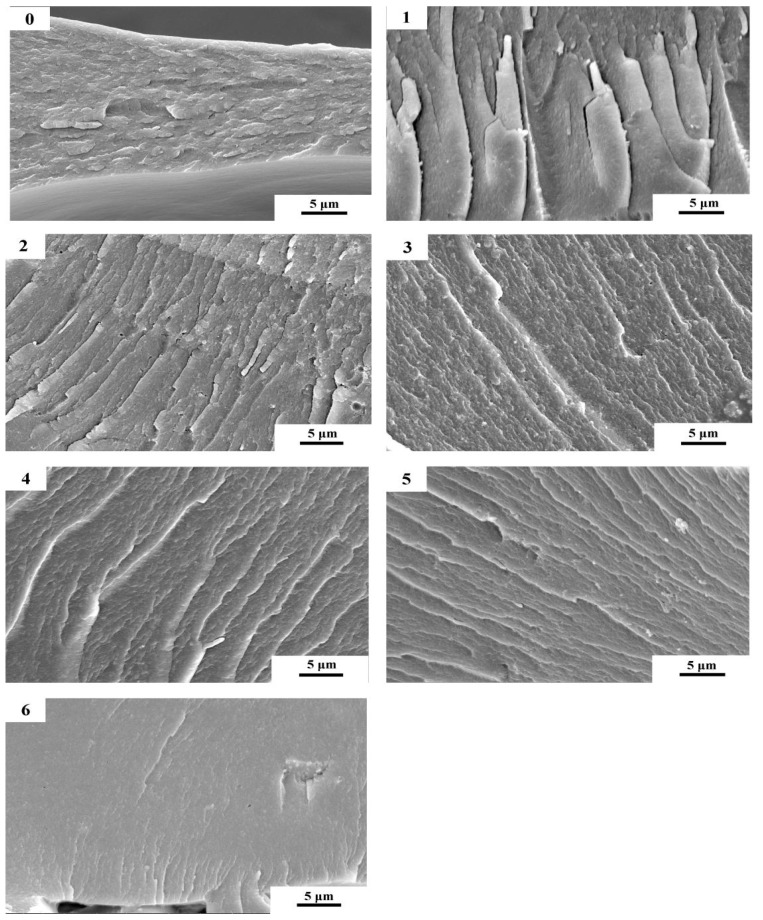
Fracture surface micrographs of the different cured adhesive samples: 0 (12 wt% SPI), 1 (18 wt% SPI/bromelain), 2 (18 wt% SPI/bromelain/3 wt% TGA), 3 (18 wt% SPI/bromelain /6 wt% TGA), 4 (18 wt% SPI/bromelain/9 wt% TGA), 5 (18 wt% SPI/bromelain/12 wt% TGA), and 6 (18 wt% SPI/9 wt% TGA).

Fracture surface micrographs of various types of cured adhesives are shown in Figure 7. SPI adhesive (0) showed a loose surface with sparse rifts. These sparse rifts could be the channels for subsequent water intrusion, reducing the water resistance of the adhesive [34]. After enzymatic hydrolysis, broken SPI molecular chains exposed more hydrophilic groups and increased the hydrophilic characteristic of adhesive (1). This occurrence resulted in a more disordered fracture surface and larger rifts, leading to a reduction in the water resistance of adhesive (1). However, with an increase in TGA addition, the cracks of enzymatic hydrolysis adhesives in the fracture surface became uniform, and the rifts disappeared gradually. These effects indicated that TGA cross-linked with soy protein molecules to increase the cross-link density, which improved the water resistance of the adhesive. Compared with adhesive (0), adhesive (6) had a smoother fracture surface and fewer cracks, indicating that TGA addition increased the brittleness of the adhesive. Enzymatic hydrolysis created a ductile fracture surface of adhesive (4) relative to adhesive (6), indicating an increase in the toughness of the adhesive, which contributed to the bond performance of the adhesive.

## 4. Conclusions

From the study on the modification of soy protein adhesives by enzymatic hydrolysis, the following conclusions were drawn:

(1) Using 0.1 wt% bromelain effectively reduced the viscosity of the SPI adhesive by 67.9% and improved the SPI content of the adhesive from 12 wt% to 18 wt%, while maintaining a similar viscosity. After the enzymatic hydrolysis process, the residual rate of the SPI/bromelain adhesive markedly decreased by 9.6%, and the wet shear strength of the resultant plywood was reduced to 70.4%. These reductions were attributed to the breakdown of the soy protein molecules into polypeptide chains and the exposure of more hydrophilic groups.
(2) With the addition of 9 wt% TGA, the residual rate of the SPI/bromelain/TGA adhesive improved by 13.7%, and the wet shear strength of the resultant plywood increased by 681.3% to 1.25 MPa, relative to that of the SPI/bromelain adhesive. This wet shear strength was 30.2% higher than that of the SPI/TGA adhesive. This improvement was attributed to the breakdown of soy protein molecules into polypeptide chains. This occurrence led to (1) the formation of more interlocks with the wood surface during the curing process of the adhesive and (2) the exposure of more hydrophilic groups and increase in the reactivity of protein with TGA, leading to a denser cross-linked network produced in the adhesive.
(3) The formed cross-linked structure exhibited a higher thermal stability after enzymatic hydrolysis, indicating an improvement in the cross-link density of the adhesive. This structure also created a ductile fracture surface of the adhesive, indicating an improvement in the toughness of the adhesive.

## Figures and Tables

**Table 1:** Various adhesive formulations. SPI—Soy protein isolate; TGA—Triglycidylamine.

Sample	SPI (g)	Distilled Water (g)	Bromelain (g)	TGA (g)
0	12	88	0	0
1	18	82	0.1	0
2	18	82	0.1	3
3	18	82	0.1	6
4	18	82	0.1	9
5	18	82	0.1	12
6	18	82	0	9

**Table 2:** Initial viscosity of different adhesive samples: 0 (12 wt% SPI), 1 (18 wt% SPI/bromelain), 2 (18 wt% SPI/bromelain/3 wt% TGA), 3 (18 wt% SPI/bromelain/6wt% TGA), 4 (18 wt% SPI/bromelain/9 wt% TGA), 5 (18 wt% SPI/bromelain /12 wt% TGA), and 6 (18 wt% SPI/9 wt% TGA).

Sample	0	1	2	3	4	5	6
Viscosity (cP)	61,000 ± 2896	62,880 ± 3263	32,293 ± 1892	2439 ± 433	707 ± 82	285 ± 57	2200 ± 387
